# DeepUMQA3: a web server for accurate assessment of interface residue accuracy in protein complexes

**DOI:** 10.1093/bioinformatics/btad591

**Published:** 2023-09-22

**Authors:** Jun Liu, Dong Liu, Gui-Jun Zhang

**Affiliations:** College of Information Engineering, Zhejiang University of Technology, Hangzhou 310023, China; College of Information Engineering, Zhejiang University of Technology, Hangzhou 310023, China; College of Information Engineering, Zhejiang University of Technology, Hangzhou 310023, China

## Abstract

**Motivation:**

Model quality assessment is a crucial part of protein structure prediction and a gateway to proper usage of models in biomedical applications. Many methods have been proposed for assessing the quality of structural models of protein monomers, but few methods for evaluating protein complex models. As protein complex structure prediction becomes a new challenge, there is an urgent need for model quality assessment methods that can accurately assess the accuracy of interface residues of complex structures.

**Results:**

Here, we present DeepUMQA3, a web server for evaluating the accuracy of interface residues of protein complex structures using deep neural networks. For an input complex structure, features are extracted from three levels of overall complex, intra-monomer, and inter-monomer, and an improved deep residual neural network is used to predict per-residue lDDT and interface residue accuracy. DeepUMQA3 ranks first in the blind test of interface residue accuracy estimation in CASP15, with Pearson, Spearman, and AUC of 0.564, 0.535, and 0.755 under the lDDT measurement, which are 17.6%, 23.6%, and 10.9% higher than the second best method, respectively. DeepUMQA3 can also assess the accuracy of all residues in the entire complex and distinguish high- and low-precision residues.

**Availability and implementation:**

The web sever of DeepUMQA3 are freely available at http://zhanglab-bioinf.com/DeepUMQA_server/.

## 1 Introduction

Predicting the three-dimensional structure of proteins is one of the major basic research issues in the field of bioinformatics, and it is important for understanding protein functions, innovative drug development, and disease treatment (Senior *et al.* 2020, Baek *et al.* 2021). Structural prediction methods typically generate many alternative models, which are then evaluated using model quality assessment procedures to select the best model and/or further guide model refinement (Liu *et al.* 2020, Zheng *et al.* 2021). Estimation of model accuracy has been an independent prediction category for CASP since 2007 (Kwon *et al.* 2021). According to the number of models used, model quality assessment (MQA) or estimation of model accuracy (EMA) methods can be divided into single-model methods (Cao *et al.* 2017, Olechnovič and Venclovas 2017, Uziela *et al.* 2017, Shuvo *et al.* 2020) and multi-model (consensus) methods (Cheng *et al.* 2019, McGuffin *et al.* 2021, Ye *et al.* 2021). The multi-model methods usually takes a model pool as the input and use information from other protein models in the model pool to evaluate the accuracy of the current model, and its performance depends largely on the number and diversity of protein models in the input model pool. The single model methods directly evaluate a single model without dependence on other models, so it has received more and more attention and research (Pages̀ *et al.* 2019, Baldassarre *et al.* 2021, Hiranuma *et al.* 2021).

In our previous work on the single-model method DeepUMQA (Guo *et al.* 2022), we designed residue-level Ultrafast Shape Recognition (USR) to effectively capture the relationship between residues and protein topology, and utilized convolutional neural networks to predict the accuracy of local residues. DeepUMQA2 (Liu *et al.* 2023b) further integrates co-evolution-based sequence features and template-based structural features to complement the shared properties between different models of the same protein, and uses an improved neural network to predict the accuracy of local residues. . As the protein monomer structure prediction problem has been largely solved, the structure prediction of protein complexes has become the new challenge (Jumper *et al.* 2021). Therefore, methods that can accurately assess the structural quality of complexes are needed (Kwon *et al.* 2021). In CASP15, the EMA category was changed from protein monomer assessment to protein complex assessment.

In this work, we proposed DeepUMQA3 web server to provide fast and accurate interface residue and per-residue accuracy prediction services for protein complexes. On the basis of previous versions, new features were designed for complex structures, and the accuracy of each residue and interface residues were predicted using an deep neural network. DeepUMQA3 ranked first in the accuracy estimation of protein complex interface residues in CASP15.

## 2 Materials and methods

**Figure 1.**
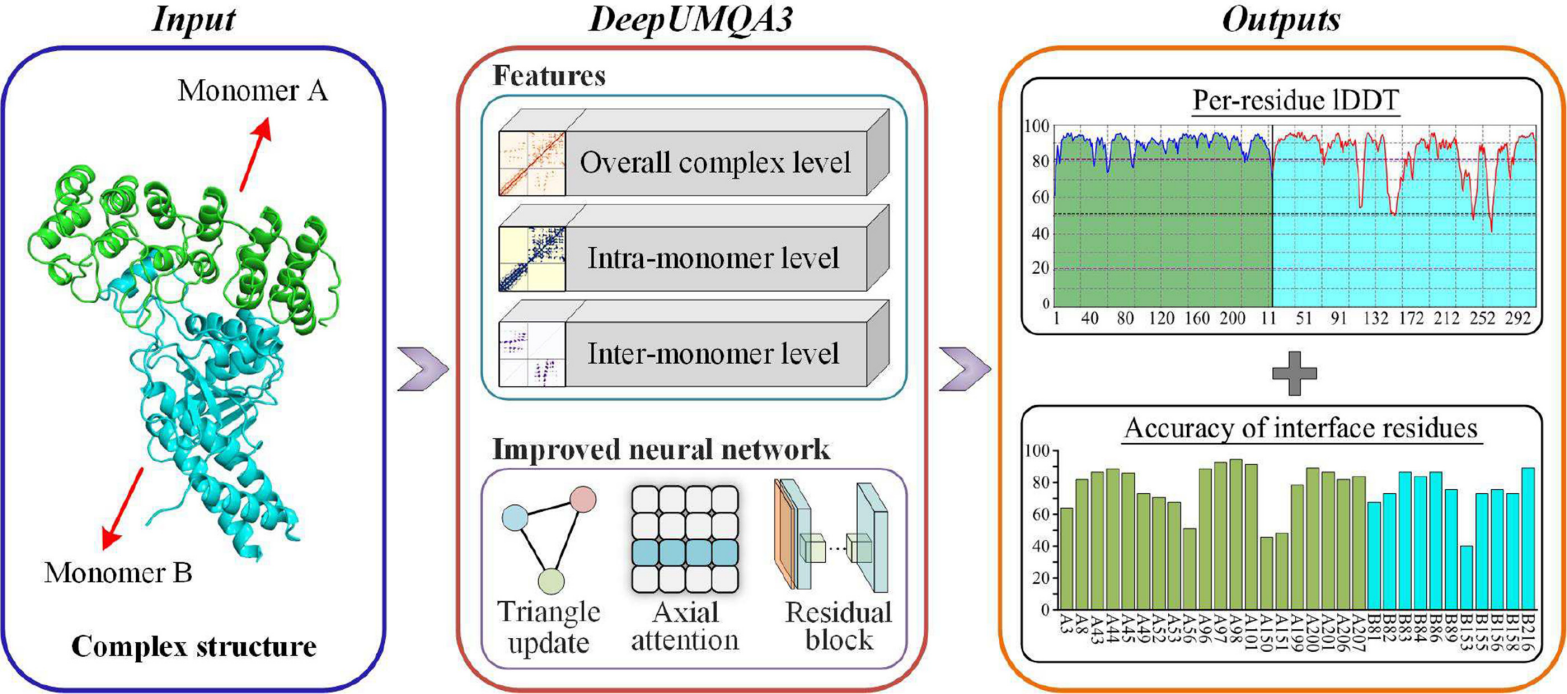
The flowchart of DeepUMQA3. For the input complex structure, it is described from three aspects: overall complex features, intra-monomer features, and inter-monomer features. Then, the extracted features are fed into a residual neural network coupled with triangle update and axial attention to predict the lDDT of each residue and the accuracy of the interface residues.

The flowchart of DeepUMQA3 is shown in Fig. 1. DeepUMQA3 describes the protein complex structures from three levels: overall complex level, intra-monomer level, and inter-monomer level. At the level of overall complex, the protein complex is regarded as a whole, and features independent of residue order were extracted, including overall USR, residue voxelization (Pages̀ *et al.* 2019), inter-residue distance and orientations (Yang *et al.* 2020), and amino acid properties (Henikoff and Henikoff 1992, Meiler *et al.* 2001). At the level of intra-monomer, the features of each monomer are extracted separately, including the sequence embedding generated by ESM-1b (Lin *et al.* 2023), secondary structure (Kabsch and Sander 1983), and Rosetta energy terms (Leaver-Fay *et al.* 2011). At the inter-monomer level, the attention map of the inter-monomer paired sequence (Lin *et al.* 2023) was used to describe the sequence relationship between monomers. In addition, inter-monomer USR is designed to describe the relationship between residues in one monomer and topologies of other monomers. The features of these three levels were fed into a convolutional neural network coupled with triangular update and axial attention to predict the per-residue lDDT (Mariani *et al.* 2013) and interface residues accuracy. A detailed description of the method can be found in the invited paper for CASP15 (Liu *et al.* 2023a).

## 3 Results

### 3.1 Performance of DeepUMQA3

DeepUMQA3 (Group name: GuijunLab-RocketX) participated in the blind test of the EMA of CASP15 and ranked first in the accuracy estimation of interface residues (see Supplementary Fig. S1). The performance of the participating methods of interface residue accuracy estimation in CASP15 is shown in Table 1.

**Table 1.** The performance of different MQA methods in the interface residues accuracy estimation blind test of CASP15.^a^^,^^b^

Method	lDDT			CAD		
	Pearson	Spearman	AUC	Pearson	Spearman	AUC
**DeepUMQA3**	**0.564**	**0.535**	**0.755**	**0.505**	**0.456**	**0.714**
ModFOLDdockS	0.455	0.416	0.674	0.420	0.379	0.660
ModFOLDdockR	0.476	0.433	0.681	0.411	0.369	0.651
VoroIF	0.333	0.339	0.664	0.272	0.271	0.619
Venclovas	0.332	0.338	0.664	0.271	0.271	0.619
FoldEver	0.277	0.279	0.625	0.217	0.194	0.583
ModFOLDdock	0.243	0.227	0.584	0.209	0.200	0.572
APOLLO	0.192	0.213	0.565	0.156	0.159	0.549
MULTICOM_deep	0.091	0.094	0.538	0.082	0.091	0.534
Manifold	0.180	0.176	0.542	0.153	0.149	0.529
DLA-Ranker	0.100	0.112	0.529	0.093	0.095	0.526
MASS	0.151	0.172	0.527	0.141	0.152	0.521
LAW	0.169	0.168	0.525	0.143	0.133	0.513

a The methods of ModFOLDdock series refer to Edmunds *et al.* (2023), the method of VoroIF refer to Olechnovič and Venclovas (2023), the method of MULTICOM_deep refer to Roy *et al.* (2023), and the other methods refer to the abstract of CASP15 (https://predictioncenter.org/casp15/doc/CASP15_Abstracts.pdf).

b Pearson and Spearman indicated the correlation between the accuracy of predicted interface residues and the real lDDT/CAD of interface residues. AUC is used to measure the ability of the MQA methods to distinguish high-/low-precision interface residues.

The bold line are the performances of our method.

DeepUMQA3’s Pearson correlation coefficient, Spearman correlation coefficient, and the area under the receiver operating characteristic curve (AUC) on lDDT (Mariani *et al.* 2013) and CAD (Olechnovič *et al.* 2013) measurements are significantly much better than those of other participating methods (see Supplementary Fig. S2). For the measurement of lDDT, DeepUMQA3’s Pearson correlation coefficient (0.564) and Spearman correlation coefficient (0.535) were 17.6% and 23.6% higher than the second ranked method, respectively, which was the only method exceeding 0.5. The AUC (0.755) of DeepUMQA3 is 10.9% higher than that of the second ranked method, indicating that DeepUMQA3 is easier to distinguish between high-/low-precision interface residues. DeepUMQA3 achieved the best performance (with highest Pearson correlation coefficient of lDDT) among the top 5 methods in 25 out of 39 targets, three of which were missed at submission and evaluated against the procedures provided by the assessor (see Supplementary Fig. S3). The performance of DeepUMQA3 on homomers and heteromers are also the best among all methods (see Supplementary Table S1). DeepUMQA3 achieved the best performance on four out of five nanobody-antigen complex targets and all three antibody–antigen complex targets, the best among all methods (see Supplementary Table S2 and Supplementary Fig. S4). DeepUMQA3 can not only assess the accuracy of interface residues, but also predict the accuracy of all residues in the entire complex, and can distinguish high-low-precision residues, which may provide important information for future structure refinement (see Supplementary Tables S3, S4 and Supplementary Fig. S4). Supplementary Figure S5 presents an example of DeepUMQA3 evaluating model accuracy on one structural model of the target T1170. It can be found that the predicted lDDT of the interface residues is very close to the real one, and the Pearson correlation coefficient, average residue-wise S-score error (ASE) (Kwon *et al.* 2021) and AUC are 0.771, 0.941 and 0.872, respectively. For all residues in the complex, the predicted lDDT can accurately capture the changing of residue accuracy, and can easily distinguish high quality and low quality regions from the overall structure. The performance of DeepUMQA3 in the overall accuracy assessment of protein complexes in CASP15 is shown in Supplementary Table S5. The DeepUMQA3 web server is suitable for interface residue accuracy assessment in protein complexes. Although the DeepUMQA3 web server provides global lDDT as a reference, methods such as MULTICOM_qa (Roy *et al.* 2023), ModFOLDdock (Edmunds *et al.* 2023), and GraphGPSM (He *et al.* 2023) are more suitable for global accuracy assessment of protein complexes.

### 3.2 The web server of DeepUMQA3

The submission page of the web server is shown in Supplementary Fig. S6. The only mandatory input is the query protein complex structure information in PDB format. Users have the option to either input complex structure data in the designated text box, or upload the PDB file of the complex structure. Additionally, they can add multiple complex structures using the “Add model” option, or upload all complex structures that need to be evaluated in a single zip file. For the complex structures submitted by users, DeepUMQA3 first extracts features, then utilizes pretrained network models to predict the accuracy of per-residue lDDT and interface residues, and finally generates a result page. If the user provides an email address, they will receive a task confirmation email after submitting the task and an email with evaluation results and web page when the task is completed. Supplementary Figure S7 demonstrates an example result web page. For each complex structure submitted by the user, the result web page will display the 3D structure, a graph of the per-residue lDDT, and the accuracy of the interface residues for each chain. Users can download each result individually, or download a compressed package of all results. Supplementary Figure S8 shows the runtime of the DeepUMQA3 web server (CPU only) on the 35 CASP15 protein complexes with a length of <3000 amino acids. Supplementary Figure S9 shows the throughput test results of the DeepUMQA3 web server.

## Supplementary Material

btad591_Supplementary_Data

## References

[btad591-B1] Baek M , DiMaio F, Anishchenko I et al Accurate prediction of protein structures and interactions using a three-track neural network. Science 2021;373:871–6. 34282049 10.1126/science.abj8754 PMC7612213

[btad591-B2] Baldassarre F , Menéndez Hurtado D, Elofsson A et al GraphQA: protein model quality assessment using graph convolutional networks. Bioinformatics 2021;37:360–6. 32780838 10.1093/bioinformatics/btaa714 PMC8058777

[btad591-B3] Cao R , Adhikari B, Bhattacharya D et al QAcon: single model quality assessment using protein structural and contact information with machine learning techniques. Bioinformatics 2017;33:586–8. 28035027 10.1093/bioinformatics/btw694 PMC6041872

[btad591-B4] Cheng J , Choe M-H, Elofsson A et al Estimation of model accuracy in CASP13. Proteins Struct Funct Bioinf 2019;87:1361–77. 10.1002/prot.25767 PMC6851425 31265154

[btad591-B5] Edmunds NS , Alharbi SMA, Genc AG et al Estimation of model accuracy in CASP15 using the ModFOLDdock server. Proteins Struct Funct Bioinf 2023; 10.1002/prot.26532 PMC10952711 37314190

[btad591-B6] Guo S-S , Liu J, Zhou X-G et al DeepUMQA: ultrafast shape recognition-based protein model quality assessment using deep learning. Bioinformatics 2022;38:1895–903. 35134108 10.1093/bioinformatics/btac056

[btad591-B7] He G , Liu J, Liu D et al GraphGPSM: a global scoring model for protein structure using graph neural networks. Brief Bioinf 2023;24:bbad219. 10.1093/bib/bbad219 37317619

[btad591-B8] Henikoff S , Henikoff JG. Amino-acid substitution matrices from protein blocks. Proc Natl Acad Sci USA 1992;89:10915–9. 1438297 10.1073/pnas.89.22.10915 PMC50453

[btad591-B9] Hiranuma N , Park H, Baek M et al Improved protein structure refinement guided by deep learning based accuracy estimation. Nat Commun 2021;12:1340. 33637700 10.1038/s41467-021-21511-x PMC7910447

[btad591-B10] Jumper J , Evans R, Pritzel A et al Highly accurate protein structure prediction with AlphaFold. Nature 2021;596:583–9. 34265844 10.1038/s41586-021-03819-2 PMC8371605

[btad591-B11] Kabsch W , Sander C. Dictionary of protein secondary structure: pattern recognition of hydroge-bonded and geometrical features. Biopolym Original Res Biomol 1983;22:2577–637. 10.1002/bip.360221211 6667333

[btad591-B12] Kwon S , Won J, Kryshtafovych A et al Assessment of protein model structure accuracy estimation in CASP14: old and new challenges. Proteins Struct Funct Bioinf 2021;89:1940–8. 10.1002/prot.26192 PMC8616788 34324227

[btad591-B13] Leaver-Fay A , Tyka M, Lewis SM et al An object-oriented software suite for the simulation and design of macromolecules. Methods Enzymol 2011;487:545–74. 21187238 10.1016/B978-0-12-381270-4.00019-6 PMC4083816

[btad591-B14] Lin Z , Akin H, Rao R et al Evolutionary-scale prediction of atomic-level protein structure with a language model. Science 2023;379:1123–30. 36927031 10.1126/science.ade2574

[btad591-B15] Liu J , Zhou X-G, Zhang Y et al CGLFold: a contact-assisted de novo protein structure prediction using global exploration and loop perturbation sampling algorithm. Bioinformatics 2020;36:2443–50. 31860059 10.1093/bioinformatics/btz943

[btad591-B16] Liu J , Liu D, He G et al Estimating protein complex model accuracy based on ultrafast shape recognition and deep learning in CASP15. Proteins Struct Funct Bioinf 2023a; 10.1002/prot.26564 37553848

[btad591-B17] Liu J , Zhao K, Zhang G et al Improved model quality assessment using sequence and structural information by enhanced deep neural networks. Brief Bioinform 2023b;24:bbac507. 36460624 10.1093/bib/bbac507

[btad591-B18] Mariani V , Biasini M, Barbato A et al lDDT: a local superposition-free score for comparing protein structures and models using distance difference tests. Bioinformatics 2013;29:2722–8. 23986568 10.1093/bioinformatics/btt473 PMC3799472

[btad591-B19] McGuffin LJ , Aldowsari FMF, Alharbi SMA et al ModFOLD8: accurate global and local quality estimates for 3D protein models. Nucleic Acids Res 2021;49:W425–30. 33963867 10.1093/nar/gkab321 PMC8218196

[btad591-B20] Meiler J , Zeidler A, Schmäschke F et al Generation and evaluation of dimension-reduced amino acid parameter representations by artificial neural networks. J Mol Model 2001;7:360–9.

[btad591-B21] Olechnovič K , Kulberkytė E, Venclovas C et al CA-score: a new contact area differenc-based function for evaluation of protein structural models. Proteins Struct Funct Bioinf 2013;81:149–62. 10.1002/prot.24172 22933340

[btad591-B22] Olechnovič K , Venclovas Č. VoroMQA: assessment of protein structure quality using interatomic contact areas. Proteins Struct Funct Bioinf 2017;85:1131–45. 10.1002/prot.25278 28263393

[btad591-B23] Olechnovič K , Venclovas Č. VoroIF-GNN: Voronoi tessellation-derived protein interface assessment using a graph neural network. Proteins Struct Funct Bioinf 2023; 10.1002/prot.26554 37482904

[btad591-B24] Pages̀ G , Charmettant B, Grudinin S. Protein model quality assessment using 3D oriented convolutional neural networks. Bioinformatics 2019;35:3313–9. 30874723 10.1093/bioinformatics/btz122

[btad591-B25] Roy RS , Liu J, Giri N et al Combining pairwise structural similarity and deep learning interface contact prediction to estimate protein complex model accuracy in CASP15. Proteins Struct Funct Bioinf 2023; 10.1002/prot.26542 PMC10749984 37357816

[btad591-B26] Senior AW , Evans R, Jumper J et al Improved protein structure prediction using potentials from deep learning. Nature 2020;577:706–10. 31942072 10.1038/s41586-019-1923-7

[btad591-B27] Shuvo MH , Bhattacharya S, Bhattacharya D et al QDeep: distance-based protein model quality estimation by residue-level ensemble error classifications using stacked deep residual neural networks. Bioinformatics 2020;36:i285–91. 32657397 10.1093/bioinformatics/btaa455 PMC7355297

[btad591-B28] Uziela K , Menéndez Hurtado D, Shu N et al ProQ3D: improved model quality assessments using deep learning. Bioinformatics 2017;33:1578–80. 28052925 10.1093/bioinformatics/btw819

[btad591-B29] Yang J , Anishchenko I, Park H et al Improved protein structure prediction using predicted interresidue orientations. Proc Natl Acad Sci USA 2020;117:1496–503. 31896580 10.1073/pnas.1914677117 PMC6983395

[btad591-B30] Ye L , Wu P, Peng Z et al Improved estimation of model quality using predicted inter-residue distance. Bioinformatics 2021;37:3752–9. 34473228 10.1093/bioinformatics/btab632

[btad591-B31] Zheng W , Zhang C, Li Y et al Folding non-homologous proteins by coupling deep-learning contact maps with I-TASSER assembly simulations. Cell Rep Methods 2021;1:100014. 34355210 10.1016/j.crmeth.2021.100014 PMC8336924

