# Supplementary material for: DeepUMQA3: a web server for accurate assessment of interface residue accuracy in protein complexes
Source: Bioinformatics. 2023 Sep 22;39(10):btad591. doi: 10.1093/bioinformatics/btad591 (PMC10560100; doi:10.1093/bioinformatics/btad591)
Supplement: btad591_Supplementary_Data [file btad591_supplementary_data.pdf]

DeepUMQA3: a web server for accurate assessment of interface residue accuracy in protein complexes

Jun Liu, Dong Liu and Guijun Zhang

Supplementary Information

Supplementary Tables

Table S1. Performance of all methods under lDDT measurement for 19 homomer targets and 20 heteromer targets in the accuracy estimation of interface residues in CASP15.

Type	Homomers (19)			Heteromers (20)		
	Pearson	Spearman	AUC	Pearson	Spearman	AUC
DeepUMQA3	0.591	0.538	0.758	0.550	0.536	0.753
ModFOLDdockR	0.517	0.466	0.698	0.436	0.401	0.664
ModFOLDdockS	0.448	0.404	0.669	0.461	0.428	0.678
ModFOLDdock	0.276	0.256	0.596	0.211	0.200	0.572
Manifold	0.181	0.155	0.531	0.179	0.195	0.551
MULTICOM_deep	0.086	0.090	0.541	0.096	0.098	0.535
Venclovas	0.341	0.336	0.665	0.322	0.341	0.662
MASS	0.130	0.162	0.529	0.175	0.182	0.525
LAW	0.114	0.123	0.539	0.235	0.221	0.509
APOLLO	0.228	0.255	0.587	0.150	0.163	0.538
VoroIF	0.343	0.337	0.665	0.322	0.341	0.662
FoldEver	0.322	0.322	0.642	0.229	0.234	0.607
DLA-Ranker	0.095	0.099	0.533	0.105	0.124	0.525

Table S2. Performance of all methods under lDDT measurement for 5 nanobody complex targets and 3 antibody-antigen complex targets in the accuracy estimation of interface residues in CASP15.

Type	Nanobody targets (5)			Antibody-antigen targets (3)		
	Pearson	Spearman	AUC	Pearson	Spearman	AUC
DeepUMQA3	0.493	0.489	0.723	0.592	0.667	0.824
ModFOLDdockR	0.377	0.33	0.619	0.338	0.402	0.699
ModFOLDdockS	0.267	0.231	0.581	0.374	0.51	0.736
ModFOLDdock	0.215	0.229	0.586	0.182	0.235	0.623
Manifold	0.197	0.22	0.528	0.128	0.186	0.571
MULTICOM_deep	0.172	0.197	0.616	0.038	0.012	0.5
Venclovas	0.103	0.116	0.549	NA	NA	NA
MASS	0.12	0.136	0.562	0.445	0.417	0.5
LAW	0.438	0.435	0.5	0.158	0.084	0.5
APOLLO	0.137	0.175	0.5	0.289	0.356	0.663
VoroIF	0.103	0.116	0.549	NA	NA	NA
FoldEver	0.206	0.21	0.595	0.245	0.274	0.637
DLA-Ranker	0.08	0.085	0.524	0.215	0.253	0.5

Table S3. Performance of DeepUMQA3 for predicting per-residue lDDT of the overall complex on different type of targets in CASP15 (Local QA level).

Type	Pearson	Spearman	Kendall	AUC	MAE
All (39)	0.625	0.551	0.395	0.823	0.138
Homomer (19)	0.617	0.555	0.401	0.818	0.157
Heteromer (20)	0.631	0.547	0.389	0.828	0.120
Nanobody (5)	0.555	0.476	0.331	0.789	0.122
Antibody-antigen (3)	0.700	0.598	0.426	0.875	0.087

Note: Local QA is calculated based on the predicted and real lDDT for each residue. Pearson, Spearman, and Kendall are used to measure the correlation of the predicted residue's lDDT with the real lDDT. AUC is used to measure the ability of the predicted lDDT to discriminate high-/low-precision residues. MAE was used to measure the difference between the predicted residue lDDT and the real lDDT.

Table S4. Performance of DeepUMQA3 for predicting per-residue lDDT of the overall complex on different type of targets in CASP15 (Global QA level).

Type	Pearson	Spearman	Kendall	AUC	MAE
All (39)	0.791	0.568	0.422	0.951	0.113
Homomer (19)	0.766	0.597	0.443	0.939	0.141
Heteromer (20)	0.814	0.542	0.402	0.963	0.085
Nanobody (5)	0.818	0.640	0.481	0.973	0.089
Antibody-antigen (3)	0.902	0.632	0.462	0.999	0.063

Note: Global QA is computed based on the global lDDT and the real lDDT of each model. Pearson, Spearman, and Kendall are used to measure the correlation of the predicted model's global lDDT with the real global lDDT. AUC is used to measure the ability of the predicted global lDDT to discriminate high-/low-precision models. MAE is used to measure the difference between the predicted model's global lDDT and the real global lDDT.

Table S5. Performance of DeepUMQA3 and other participating methods for overall complex accuracy evaluation measured by TM-score and global lDDT in CASP15.

Method	TM-score				Global lDDT			
	Pearson	Spearman	AUC	Loss	Pearson	Spearman	AUC	Loss
DeepUMQA3	0.464	0.361	0.655	0.165	0.781	0.568	0.741	0.058
MULTICOM_qa	**0.712**	**0.580**	**0.703**	0.152	0.64	0.582	0.713	0.068
MULTICOM_deep	0.183	0.176	0.561	0.299	0.179	0.187	0.58	0.198
MULTICOM_egnn	0.257	0.257	0.607	0.201	0.323	0.332	0.627	0.127
ModFOLDdockS	0.551	0.434	0.645	0.227	0.719	0.619	0.729	0.111
ModFOLDdockR	0.635	0.504	0.679	0.142	**0.829**	0.688	0.762	0.063
ModFOLDdock	0.636	0.517	0.684	0.127	0.776	**0.694**	**0.766**	**0.054**
Venclovas	0.490	0.437	0.680	**0.123**	0.48	0.543	0.732	**0.054**
VoroIF	0.483	0.351	0.641	0.160	0.469	0.416	0.675	0.097
VoroMQA-select-2020	0.414	0.394	0.663	0.179	0.502	0.514	0.706	0.097
Manifold	0.541	0.510	0.664	0.146	0.526	0.541	0.706	0.097
MASS	0.269	0.165	0.566	0.259	0.42	0.241	0.577	0.164
GuijunLab-Assembly	0.437	0.373	0.630	0.193	0.722	0.6	0.734	0.085
GuijunLab-Human	0.601	0.459	0.640	0.189	0.557	0.476	0.671	0.085
GuijunLab-Threader	0.633	0.493	0.668	0.207	0.569	0.48	0.66	0.114
APOLLO	0.059	0.056	0.510	0.247	0.069	0.053	0.507	0.144
Bhattacharya	0.474	0.423	0.655	0.195	0.717	0.562	0.708	0.08
ChaePred	0.453	0.327	0.651	0.233	0.448	0.33	0.65	0.208
FoldEver	0.381	0.247	0.594	0.267	0.451	0.3	0.622	0.161
MUFold	0.625	0.409	0.617	0.240	0.553	0.318	0.597	0.17
MUFold2	0.564	0.351	0.604	0.239	0.48	0.333	0.606	0.149
LAW	0.332	0.215	0.595	0.305	0.512	0.323	0.63	0.205

Note: Pearson and Spearman are used to measure the correlation of the predicted model's global accuracy with the real global accuracy. AUC reflects the ability of the model quality evaluation method to distinguish between high-accuracy and low-accuracy models. Loss represents the difference between the selected best model based on evaluation scores and the real best model, reflecting the ability of the model quality evaluation method to select the best model.

Supplementary Figures

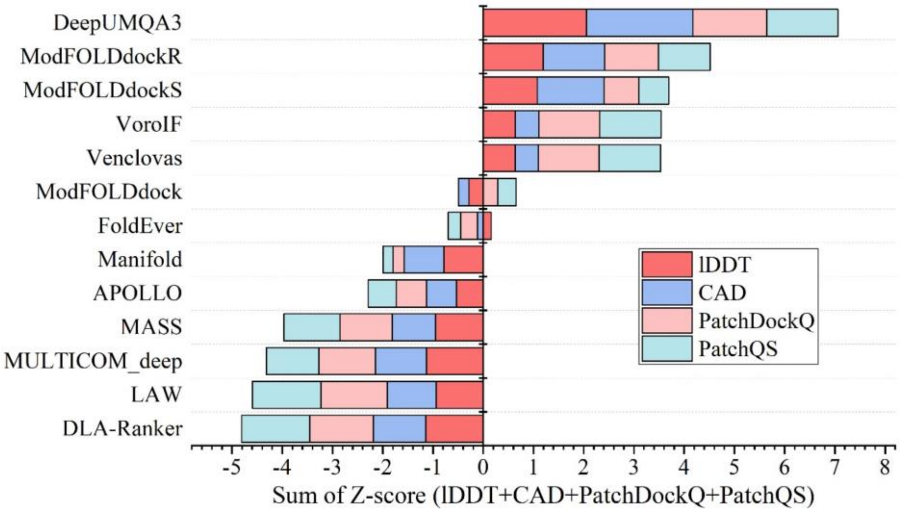

Figure S1. Ranking of the methods for interface residue precision estimation in CASP15 according to the sum of average Z-scores for lDDT (Red), CAD (blue), PatchDockQ (light red) and PatchQS(cyan). The Z-score of each item is weighted according to the Z-score of Pearson, Spearman and AUC according to the weight of 0.1:0.5:1. The data comes from the CASP15 official website (https://predictioncenter.org/casp15/qa_local.cgi). The group name of DeepUMQA3 in CASP15 is "GuijunLab-RocketX".

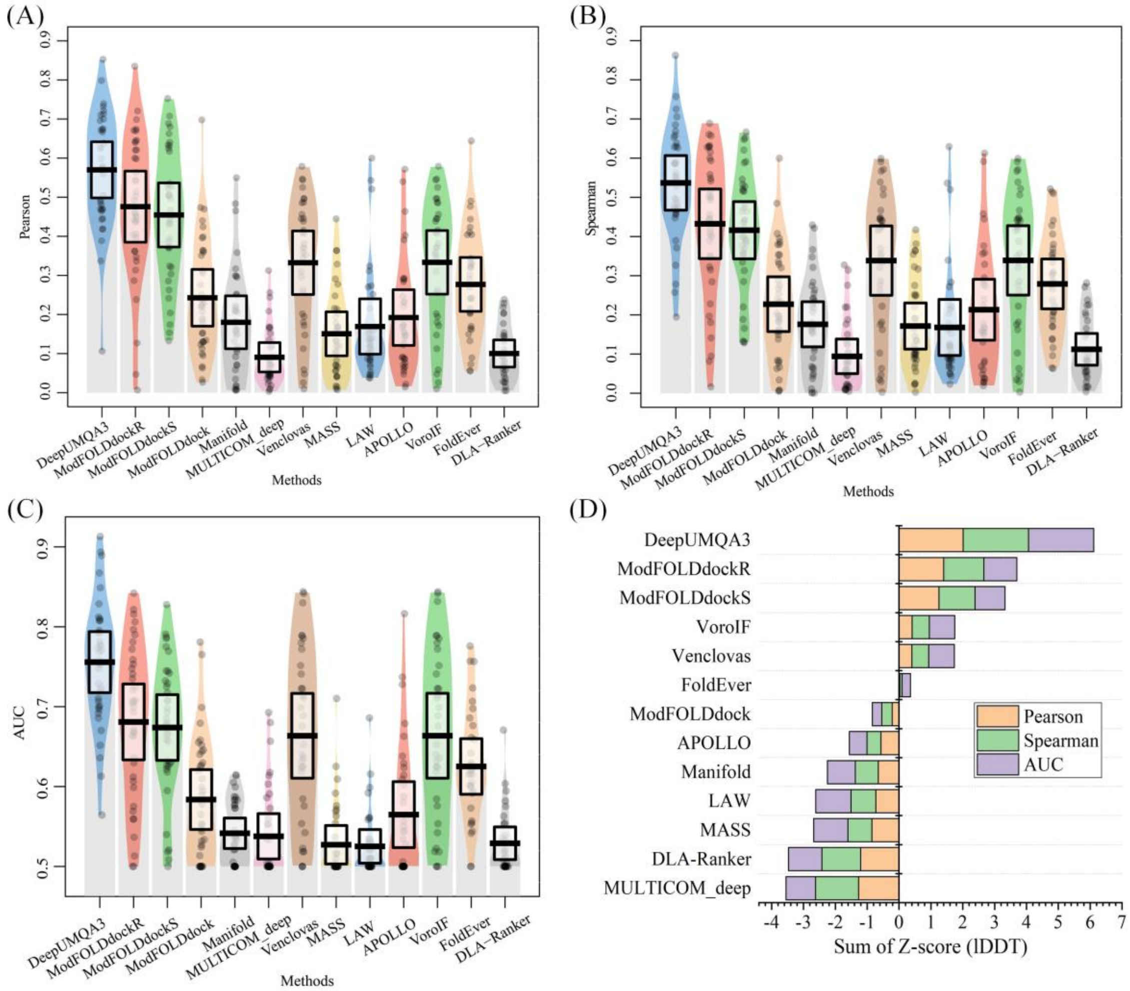

Figure S2. Performance of methods for assessing the accuracy of interface residues under lDDT measurement in CASP15. (A), (B), and (C) are the pirate graphs of Pearson, Spearman, and AUC for all participating methods on 39 targets, respectively. (D) is the ranking of Z-score of Pearson, Spearman and AUC.

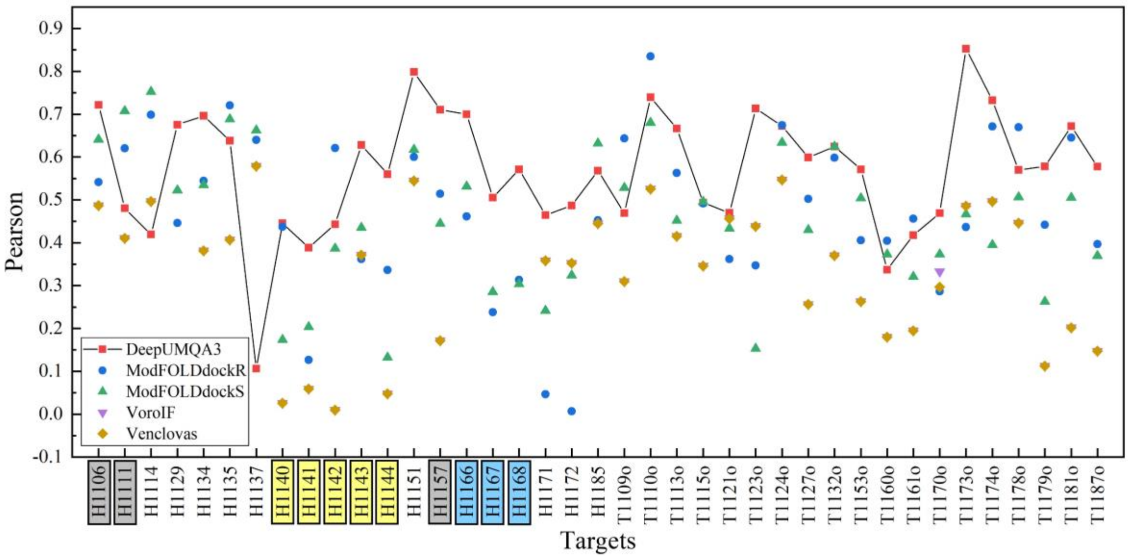

Figure S3. The Pearson correlation coefficient under lDDT measurement of the top 5 methods for interface residue accuracy evaluation on the 39 targets in CASP15. The targets in the gray boxes are the targets that were missed when DeepUMQA3 was submitted the results, and we evaluated them using the programs provided by the assessor. The target in the yellow box is the nanobody complex, and the target in the blue box is the antibody-antigen complex.

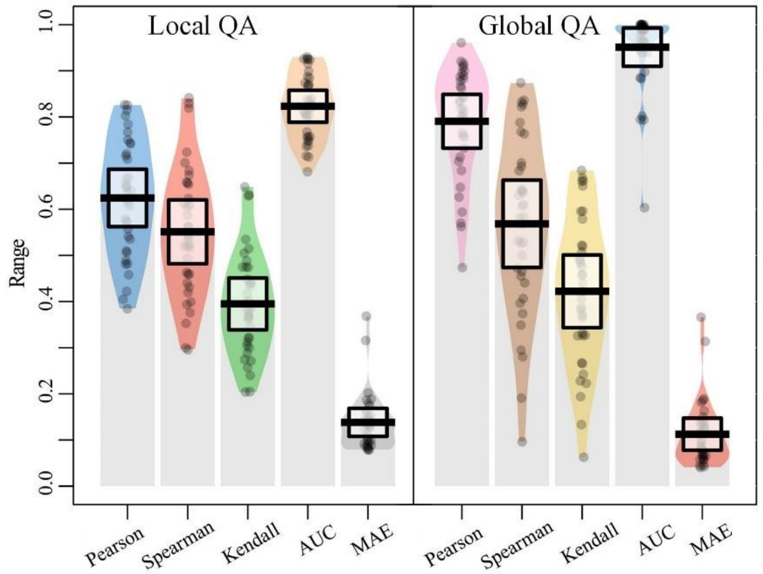

Figure S4. Pirate graphs on different performance indicators of DeepUMQA3 for predicting per-residue lDDT of overall complex in CASP15. On the left is the pirate graph of Local QA, and on the right is the pirate graph of Global QA. The higher the Pearson, Spearman, and Kendall, the stronger the correlation between the predicted lDDT and the real lDDT. The higher the AUC, the stronger the ability of DeepUMQA3 to distinguish high-/low-precision residues/models. The smaller the MAE, the difference in lDDT is smaller.

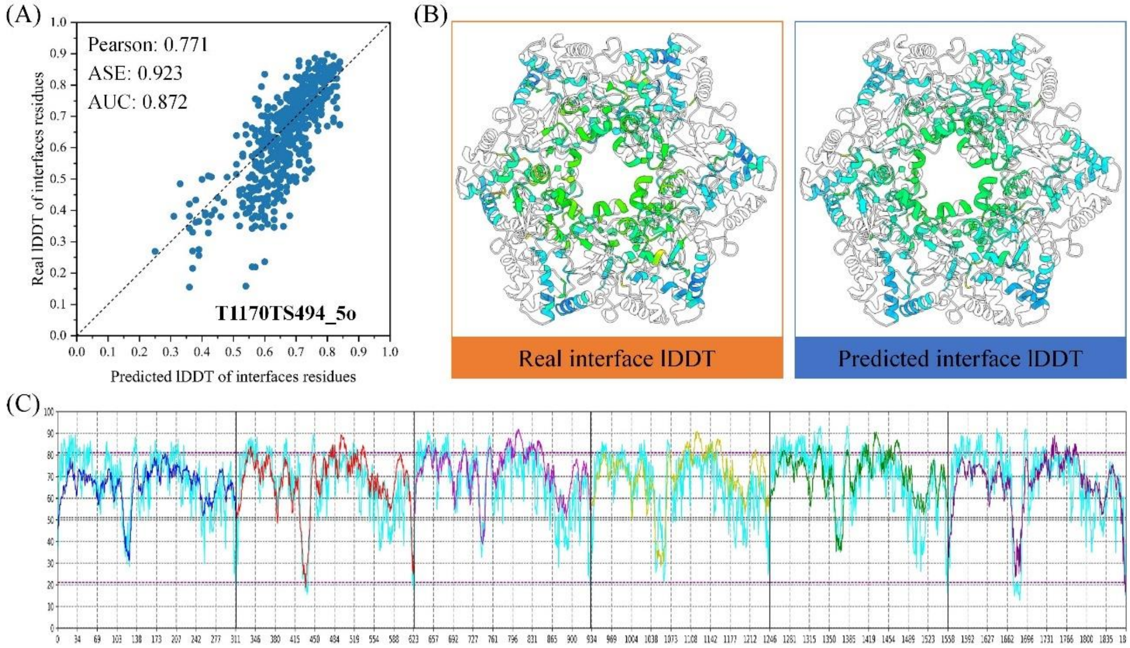

Figure S5. An example of DeepUMQA3 evaluating the model on the structural model T1170TS494_5o of the target T1170. T1170 is a homomer composed of 6 identical monomer structures, containing 1908 residues. (A) Head-to-head comparison of the predicted interface residue lDDT with the real lDDT. (B) The real interface residue lDDT (left) and the predicted interface residue lDDT (right) in the structural model. The colored parts represent the interface residues, and red to blue represents lDDT from 0 to 100. (C) Real lDDT (cyan) and predicted lDDT (other colors) for all residues in the overall protein complex, with predicted lDDT for different monomers indicated in different colors.

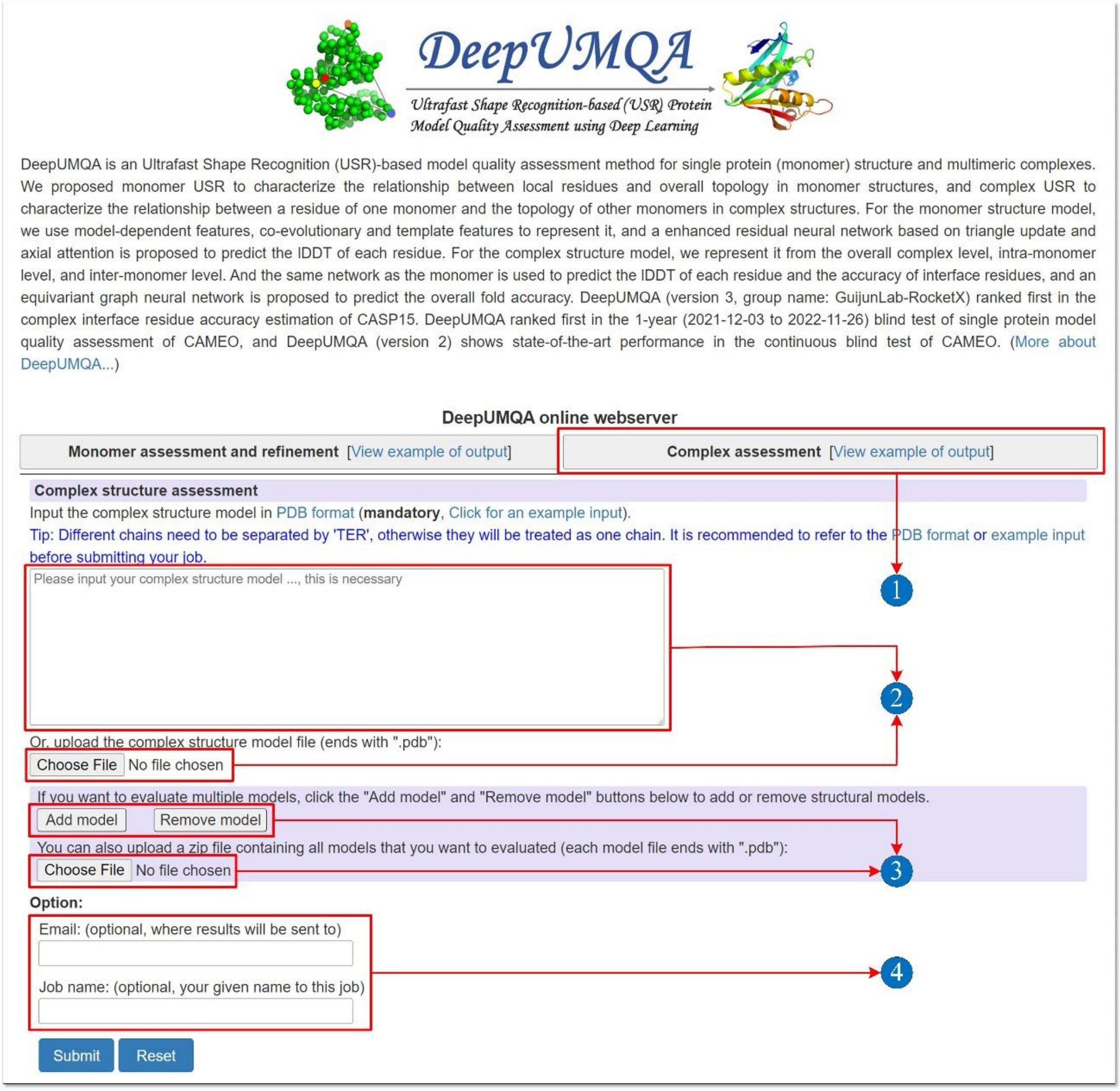

Figure S6. DeepUMQA3 server job submission. The DeepUMQA3 server is integrated into the DeepUMQA server, and the submission of complex model quality assessment tasks can be started by clicking the “Complex assessment” button (1). Users can enter the model data of the complex structure through the text box or upload the PDB file (2). Users can add multiple complex structures for evaluation via the "Add model" and "Remove model" buttons or upload all complex structures that need to be evaluated in a single zip file (3). Users can optionally provide an “Email” to receive result notifications and a “Job name” (4). Submit or reset tasks via the “Submit” or “Reset” buttons.

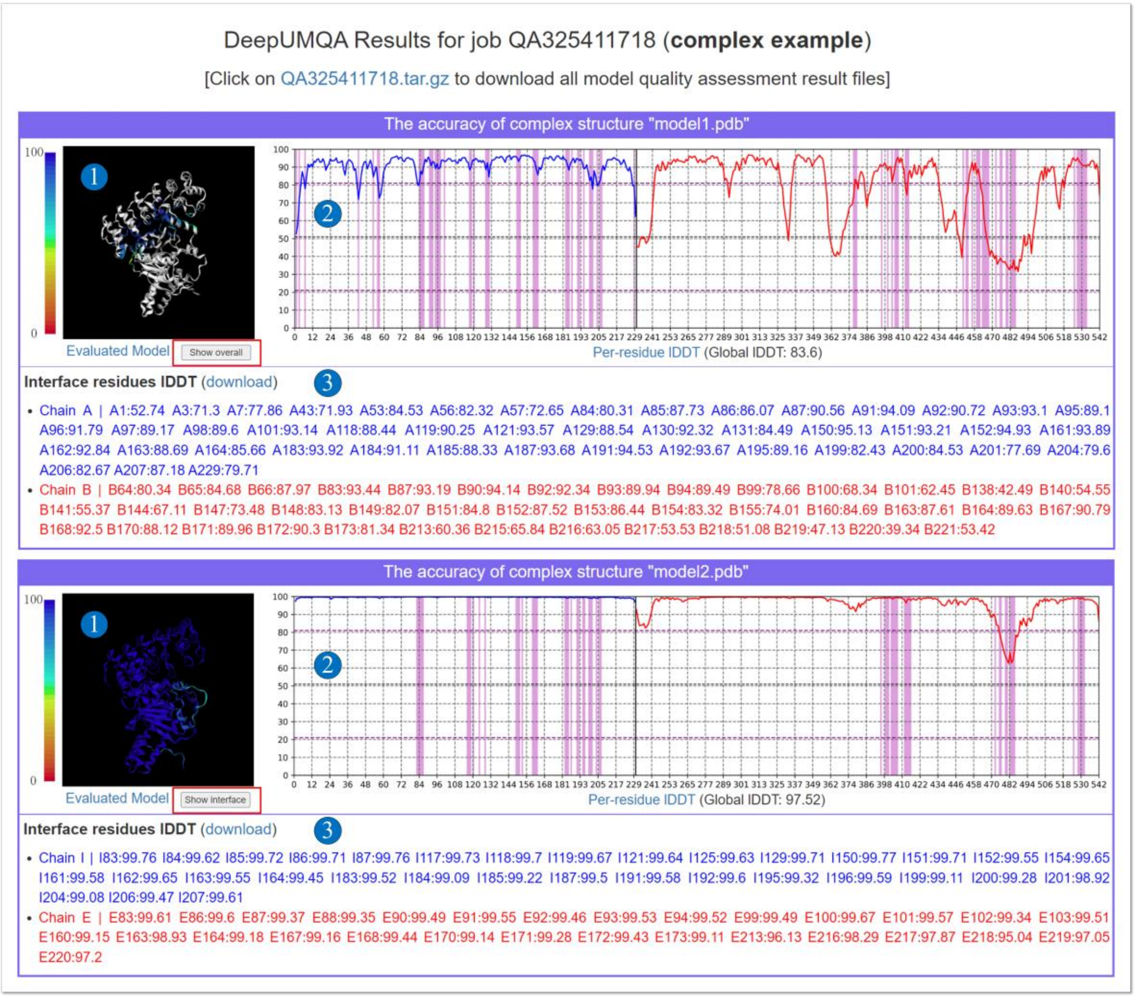

Figure S7. Example of DeepUMQA3 result web page. For each evaluated complex model, the results page displays three aspects. (1) The model structure with color markings, where the color ranges from red to blue, indicating the lDDT scores of residues from 0 to 100. Users can view the overall structure or interface residue structure by clicking on the “Show interface”/ “Show overall” button. (2) The lDDT curves of all residues in the whole complex, the curves of different chains have different colors; the residues marked with plum color bars in the figure are interface residues. (3) The interface residues precision of all chains, different chains are represented by different colors. Users can download each result individually or download a compressed package of all results.

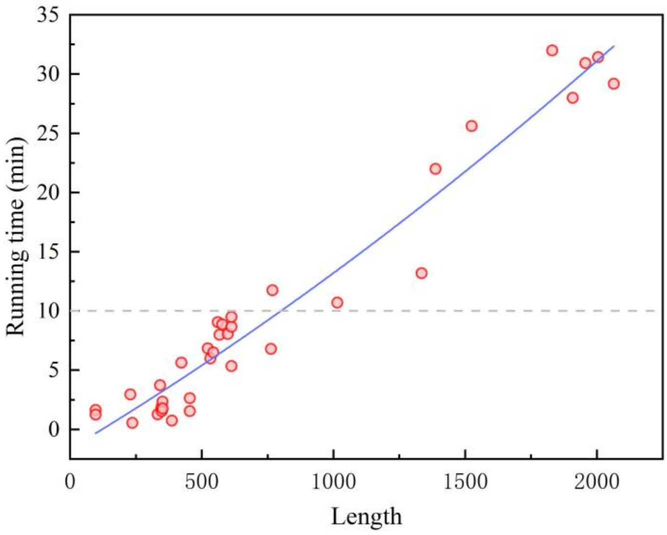

Figure S8. The running time of the DeepUMQA3 web server (CPU only) on the 35 CASP15 protein complexes with a length of less than 3,000 amino acids. In summary, for protein complexes with a length of less than 750 amino acids, the runtime is within 10 minutes. For complexes with a length of around 1000 amino acids, the runtime does not exceed 15 minutes. And for complexes with a length of around 2000 amino acids, the runtime is approximately 30 minutes.

```
D:\SoftWare Documents\Apache\Apache24\bin>abs.exe -n 100 -c 10 http://zhanglab-bioinf.com/DeepUMQA_server/
This is ApacheBench, Version 2.3 <$Revision: 1903618 $>
Copyright 1996 Adam Twiss, Zeus Technology Ltd, http://www.zeustech.net/
Licensed to The Apache Software Foundation, http://www.apache.org/

Benchmarking zhanglab-bioinf.com (be patient).....done


Server Software:        nginx/1.9.9
Server Hostname:        zhanglab-bioinf.com
Server Port:            80

Document Path:          /DeepUMQA_server/
Document Length:        26475 bytes

Concurrency Level:      10
Time taken for tests:   10.597 seconds
Complete requests:      100
Failed requests:        0
Total transferred:      2671000 bytes
HTML transferred:       2647500 bytes
Requests per second:    9.44 [#/sec] (mean)
Time per request:       1059.719 [ms] (mean)
Time per request:       105.972 [ms] (mean, across all concurrent requests)
Transfer rate:          246.14 [Kbytes/sec] received

Connection Times (ms)
              min  mean[+/-sd] median   max
Connect:       84  101   6.5    102     118
Processing:   220  884 153.2    921    1042
Waiting:      103  454 231.9    422     907
Total:        318  985 152.9   1021    1138
```

Figure S9. The results of testing the throughput of the DeepUMQA3 web server using ApacheBench. We simulated the situation where 10 users access the DeepUMQA3 network server 100 times at the same time (each user visits 10 times). The results show that all 100 visits were successful (Failed requests: 0), and the total time consumed (Time taken for tests) was 10.597 seconds. The average user access time (Time per request) and the web server average access time (Time per request, across all concurrent requests) are 1059.719ms and 105.972ms respectively. The throughput rate (Requests per second) is 9.44 times per second.
